# Cost-effectiveness of electroconvulsive therapy compared to repetitive transcranial magnetic stimulation for treatment-resistant severe depression: a decision model

**DOI:** 10.1017/S0033291714002554

**Published:** 2014-10-30

**Authors:** L. Vallejo-Torres, I. Castilla, N. González, R. Hunter, P. Serrano-Pérez, L. Perestelo-Pérez

**Affiliations:** 1 Departamento de Economía de las Instituciones, Estadística Económica y Econometría, Universidad de la Laguna, Spain; 2 Centro de Investigaciones Biomédicas de Canarias (CIBICAN), Spain; 3 Red de Investigación en Servicios de Salud en Enfermedades Crónicas (REDISSEC), Spain; 4 Servicio de Evaluación del Servicio Canario de la Salud (SESCS), Tenerife, Spain; 5 Fundación Canaria de Investigación y Salud (FUNCIS), Spain; 6 Research Unit Hospital Galdakao-Usansolo, Galdakao, Bizkaia, Spain; 7 Research Department of Primary Care and Population Health, University College London, UK; 8 Hospital Universitario de la Princesa, Madrid, Spain

**Keywords:** Cost-effectiveness, decision model, depression, electroconvulsive therapy, repetitive transcranial magnetic stimulation

## Abstract

**Background:**

Electroconvulsive therapy (ECT) is widely applied to treat severe depression resistant to standard treatment. Results from previous studies comparing the cost-effectiveness of this technique with treatment alternatives such as repetitive transcranial magnetic stimulation (rTMS) are conflicting.

**Method:**

We conducted a cost-effectiveness analysis comparing ECT alone, rTMS alone and rTMS followed by ECT when rTMS fails under the perspective of the Spanish National Health Service. The analysis is based on a Markov model which simulates the costs and health outcomes of individuals treated under these alternatives over a 12-month period. Data to populate this model were extracted and synthesized from a series of randomized controlled trials and other studies that have compared these techniques on the patient group of interest. We measure effectiveness using quality-adjusted life years (QALYs) and characterize the uncertainty using probabilistic sensitivity analyses.

**Results:**

ECT alone was found to be less costly and more effective than rTMS alone, while the strategy of providing rTMS followed by ECT when rTMS fails is the most expensive and effective option. The incremental cost per QALY gained of this latter strategy was found to be above the reference willingness-to-pay threshold used in these types of studies in Spain and other countries. The probability that ECT alone is the most cost-effective alternative was estimated to be around 70%.

**Conclusions:**

ECT is likely to be the most cost-effective option in the treatment of resistant severe depression for a willingness to pay of €30 000 per QALY.

## Introduction

Electroconvulsive therapy (ECT) is a technique widely applied to treat severe depression in patients who do not respond to treatment with medication or psychological therapies. This technique induces a controlled generalized seizure in the central nervous system through electrical stimulation. Although there is variation across countries and hospitals, ECT is now generally performed under anaesthesia, with myorelaxation, artificial ventilation, and using computerized devices to achieve an adequate seizure, safely and effectively, minimizing potential adverse events. ECT is, however, still associated with some side-effects such as amnesia (temporary or permanent), confusion, headache, and nausea (see reviews by Carney *et al.* 2003; Dunne & McLoughlin, 2012). Repetitive transcranial magnetic stimulation (rTMS) is an alternative technique more recently introduced which has the advantage of not requiring anaesthesia, it stimulates the brain non-convulsively and the reported side-effects have been limited to moderate headaches. However, some studies have found that rTMS is not as effective as ECT in the treatment of severe depression (Berlim *et al.* 2013).

In the widespread context of scarce healthcare resources, there is a need to compare healthcare strategies, not only in terms of clinical effectiveness, but also in terms of their economic consequences. This comparison provides decision makers with additional evidence to assist them in making choices between competing alternatives within budget constraints.

The cost-effectiveness of ECT *v.* rTMS has been considered in previous published studies but with conflicting results. Kozel *et al.* (2004) used a decision model to evaluate the cost-effectiveness of ECT alone, rTMS alone and rTMS followed by ECT when rTMS failed. They concluded that rTMS alone offered a considerable economic benefit on healthcare and patient costs over ECT alone, and that rTMS followed by ECT was the most effective and least costly option. Knapp *et al.* (2008) conducted an economic evaluation alongside a more recent clinical trial comparing patients randomly treated with ECT and rTMS with a 6-month follow-up (for further details on the trial see Eranti *et al.* 2007). Contrary to the results found in Kozel *et al.* (2004), they found that rTMS had a very low probability of being cost-effective compared with ECT; rTMS was found not to be as effective as ECT and there were generally no differences on healthcare costs, while informal care costs were higher with rTMS.

The aim of this study is to develop a decision analytical model of the cost-effectiveness of ECT *v.* rTMS for treatment-resistant severe depression using all relevant studies with best available quality (NICE, 2013). We do this by synthesizing all available information into a decision model that combines data from, alongside other sources, the series of randomized controlled trials that have compared ECT and rTMS in the treatment of resistant severe depression.

## Method

We conducted an economic evaluation comparing ECT with rTMS for severe depression in patients who do not respond to pharmacological and psychological therapies. In particular, and following the study published by Kozel *et al.* in 2004, we compared three alternatives: ECT alone, rTMS alone and rTMS followed by ECT when rTMS fails. The characteristics of the population of interest for this evaluation are similar to the sample characteristics defined in a recent systematic review with meta-analysis of RCTs comparing ECT *v.* rTMS (Berlim *et al.* 2013), i.e. subjects aged between 18 and 75 years with a diagnosis of (unipolar or bipolar) major depression starting treatment with ECT or rTMS without new antidepressant therapy. The primary reason for administration of ECT or rTMS treatment in these studies was resistance to standard treatment or refractoriness of depression.

The analysis took the perspective of the National Health Service (NHS) in Spain, i.e. we considered the costs that are incurred by the Spanish NHS. Effectiveness was measured using quality-adjusted life years (QALYs). QALYs are a measure of health-related quality of life (QoL) that combines information on QoL and length of life and that is widely recommended in economic evaluations to facilitate comparable decisions about resource allocation across different health conditions (NICE, 2013). QoL values for depression have been estimated in a number of studies in the literature (see review by Peasgood *et al.* 2012).

The cost-effectiveness analysis was based on a Markov model which defines the health and treatment states and possible consequences of the interventions. The prognosis of patients was modelled based on a set of possible transitions between these states over a series of discrete time periods (each cycle was defined as 15 days in our model, i.e. patients might transit from one state to another every 15 days). This model structure is more flexible than other model alternatives, such as decision trees, and allows us to easily incorporate potential events such as relapses and recurrences. Similarly to other economic analyses of depression treatment (e.g. Greenhalgh *et al.* 2005), we considered a time-frame of 12 months for the analysis, as valid data for longer periods are not readily available; hence discounting was not undertaken.

Cost-effectiveness was summarized by the incremental cost-effectiveness ratio (ICER), which is defined as the incremental cost divided by the incremental effectiveness of two competing alternatives (Drummond *et al.* 2005). The ICER represents the additional cost of one unit of outcome gained (in our case QALY) by a healthcare intervention or strategy, when compared to the next best alternative. This outcome is then compared with the decision maker's willingness-to-pay threshold per unit of effectiveness in order to draw conclusions about whether or not the intervention is viewed as cost-effective. In Spain there is not an explicit willingness-to-pay threshold, but a reference value has been estimated as €30 000/QALY (Sacristán *et al.* 2002).

We undertook a deterministic analysis using the mean values for each model parameter, and developed a probabilistic sensitivity analysis in order to characterize the uncertainty in the model results. We represented graphically the uncertainty in the results by the means of the cost-effectiveness plane and the cost-effectiveness acceptability curves (CEACs). CEACs indicate the probability that an intervention is cost-effective for different values of willingness to pay per effectiveness unit.

### Model structure

**Fig. 1.**
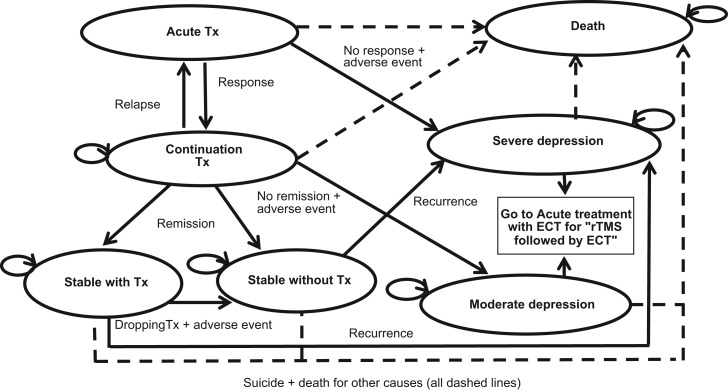
Markov model structure. Tx, treatment; ECT, Electroconvulsive therapy; rTMS, repetitive transcranial magnetic stimulation.

Fig. 1 shows the structure of the Markov model. The following specific terms are used to describe our model: response, remission, relapse, and recurrence. The definitions of these outcomes were based on those used on the studies identified and are defined as follows: patients are deemed to have responded to acute treatment when they experience a ⩾50–60% (depending on the study) decrease from baseline to end of acute treatment on the Hamilton Depression Rating Scale (HAMD); the remission criterion requires a HAMD score of ⩽8 after a period of 6 months; relapse is defined as a return of depressive symptomatology with a HAMD score of⩾16 during the continuation treatment; while we defined recurrence when the patient returns to a HAMD score of⩾16 after having remitted.

In our model a patient suffering from an acute episode of severe depression, which fails to improve after medication and/or psychological therapies, receives acute treatment for a 2-week period using ECT in the ECT-alone group or rTMS in the rTMS and rTMS + ECT groups. After the acute treatment, the patient might respond and will then move into the continuation treatment state with rTMS or ECT depending on the group, or the patient might suffer an adverse event that leads to discontinuation of the therapy or might not respond to treatment. In the last two cases, the patient will return to a health state of severe depression since their condition would have not improved. Note that we do not explicitly consider the impact on the patients of the possible adverse events separately, but we implicitly account for them by considering that patients suffering these side-effects will discontinue treatment and return to the severe depression state, which is related to a lower QoL and higher healthcare costs.

If the patient responds to the acute treatment, the continuation treatment might last for up to 6 months (with a minimum of 4 months for which we included a series of so-called ‘tunnel-states’ in the Markov model – not shown in Fig. 1). Tunnel states are additional states that facilitate accounting for time-dependency in a Markov model (Briggs *et al.* 2006). In our case, the minimum time individuals receive continuation treatment before they are classified, or not, as in remission is 4 months, but the cycle length of the model is of 15 days. Therefore, the tunnel states allow us to force individuals to stay in the continuation treatment state during at least 4 months before moving to the remission or depression states. However, at any time during the continuation treatment the patient might experience an adverse event or might relapse. In the latter case the patient will be provided with acute treatment again. After continuation treatment, the patient might be classified as in remission or might not remit and thus move to a health state of moderate depression. Those who are in remission might quit or continue with treatment and, in any case, the patient might experience a relapse and move to the severe depression state. In the case of the strategy defined as ‘rTMS followed by ECT when rTMS fails’, if patients do not respond or do not remit, or if they experience an adverse effect or a re-occurrence, they will consequently be treated with ECT as second-line treatment. At any given time and health state, there is a risk of mortality due to suicide or due to other reasons.

## Data

The data required to populate the model fall within four general types: transition probabilities between states for ECT and rTMS (and associated relative risks); use of healthcare resources for each health state and intervention; the unit costs of healthcare resources; and utility weights for the health states identified in the model to calculate QALYs.

We conducted a series of structured literature reviews using the following data sources: OvidSP, Medline, EMBASE, and the databases DARE, NHSEED and HTA at the Centre for Reviews and Dissemination at the University of York, with the primary aim of identifying RCTs comparing both interventions on the patient group of interest, as well as guidelines of clinical practice for each intervention and sources for unit costs and QALY weights. The structured literature reviews were complemented with manual searches in reference lists of identified sources, as well as Google searches and discussion with clinical experts in the field.

### Transition probabilities and relative risks

There are a number of RCTs which have compared ECT *v.* rTMS in treatment-resistant depression. A recent systematic review with meta-analysis identified seven RCTs (Berlim *et al.* 2013). Of the seven randomized trials on rTMS *v.* ECT for depression resistant to standard treatment included in this meta-analysis, six were selected for our economic evaluation (Grunhaus *et al.* 2000, 2003; Janicak *et al.* 2002; Rosa *et al.* 2006; Eranti *et al.* 2007; Keshtkar *et al.* 2011). We did not include information from one study (Pridmore *et al.* 2000) because it did not report any of the parameters of interest for our model as indicated in the model structure; but we included an additional study reporting long-term outcomes (Dannon *et al*. 2002).

With respect to the findings of the included trials, Janicak *et al.* (2002), Grunhaus *et al.* (2003), and Rosa *et al.* (2006) found no differences on response rate between ECT and rTMS, while Eranti *et al.* (2007) and Grunhaus *et al.* (2000) found a significantly higher probability of response on patients treated with ECT. Keshtkar *et al.* (2011) also found better efficacy with ECT than rTMS but did not report a response or remission rate. Dannon *et al.* (2002) reported similar probabilities of longer-term remission for both techniques, and Eranti *et al.* (2007) also reported similar probabilities of remission in the patient who responded to initial acute treatment. Rosa *et al.* (2006) reported higher rates of discontinuation of therapy due to adverse events with ECT than with rTMS while this probability was the same in other studies (Janicak *et al.* 2002; Eranti *et al.* 2007; Keshtkar *et al.* 2011).

Information required for some parameters were not available from these comparative studies, and thus were extracted from studies based on ECT assuming the same value for rTMS. These parameters were: suicide rates for individuals treated with ECT extracted from Hunt *et al.* (2011); re-occurrence of depression probability with and without treatment which was based on a study by Sackeim *et al.* (2001) focused on patients on continuation treatment after ECT; and the probability of quitting treatment during stabilization which was extracted from Dannon *et al.* (2002) (who did not differentiate by treatment arm for this parameter) and Sackeim *et al.* (2001).

For those parameters for which information was available on more than one study we conducted meta-analyses using fixed effect models to synthesize the information. As previously mentioned, the cycle length in the Markov model was 15 days. Therefore, transition probabilities and relative risks which were reported for a different time period were converted to instantaneous rates assuming a fixed rate and then probabilities for a 15-day period (see Table 1).

**Table 1.** Transition probabilities and relative risks (15-day cycle)

	Value	s.e.	Sources	Probability distribution
Transition probabilities (15-day cycle)				
Response (ECT)	0.3722	0.0521	Eranti *et al.* (2007), Rosa *et al.* (2006), Grunhaus *et al.* (2003), Janicak *et al.* (2002), Grunhaus *et al.* (2000)	Beta
Adverse effect leading to discontinuing acute treatment (ECT)	0.0549	0.0239	Janicak *et al.* (2002), Rosa *et al.* (2006), Eranti *et al.* (2007), Keshtkar *et al.* (2011)	Beta
Relapse (ECT)	0.0184	0.0301	Dannon *et al.* (2002)	Beta
Remission (at 4th month – ECT)	0.3700	0.1394	Eranti *et al.* (2007)	Beta
Remission (after 4th month – ECT)	0.0561	0.0664	Eranti *et al.* (2007)	Beta
Drop-out treatment after remission	0.0090	0.0084	Sackeim *et al.* (2001), Dannon *et al.* (2002)	Beta
Recurrence with treatment	0.0405	0.0411	Sackeim *et al.* (2001)	Beta
Recurrence without treatment	0.1416	0.0697	Sackeim *et al.* (2001)	Beta
Suicide	0.00003	0.00008	Hunt *et al.* (2011)	Beta
Death (other causes)	0.0002	–	INE	
	Value	Variance Ln (RR)	Sources	Probability distribution
Relative risks, rTMS *v*. ECT				
Response	0.7012	0.2384	Eranti *et al.* (2007), Rosa *et al.* (2006), Grunhaus *et al.* (2003), Janicak *et al.* (2002), Grunhaus *et al.* (2000)	Log normal
Adverse effect	0.8006	0.5843	Janicak *et al.* (2002), Rosa *et al.* (2006), Eranti *et al.* (2007), Keshtkar *et al.* (2011)	Log normal
Relapse	0.9474	0.4024	Dannon *et al.* (2002)	Log normal
Remission	1.0000	0.5670	Eranti *et al.* (2007)	Log normal

s.e., Standard error; ECT, electroconvulsive therapy; INE, Institute of National Statistics (in Spanish); RR, relative risk; rTMS, repetitive transcranial magnetic stimulation.

### Healthcare resource use and unit costs

Information of the use of healthcare resources for each health state and intervention were obtained from publications on current clinical practice in Spain (Bertolín-Guillén *et al*. 2006; Martínez-Amorós *et al.* 2012), from the description of resources recorded alongside a clinical trial (Knapp *et al.* 2008), and by communication with clinical experts (see Table 2).

**Table 2.** Resource use (15-day cycle), unit costs and QALY weights

		Value	s.e.	Sources	Probability distribution
Resource use (15-day cycle)					
Acute Tx	Number of sessions (ECT)	4.00	0.80	Bertolín-Guillén *et al.* (2006), Martínez-Amorós *et al.* (2012)	Gamma
	Number of sessions (rTMS)	10.00	2.00	Knapp *et al.* (2008)	Gamma
Continuation Tx	Number of sessions (ECT)	1.00	0.50	Martínez-Amorós *et al.* (2012)	Gamma
	Number of sessions (rTMS)	2.00	1.00	Expert opinion	Gamma
	In-hospital days (ECT)	0.43	0.09	Knapp *et al.* (2008)	Gamma
	In-hospital days (rTMS)	0.97	0.19	Knapp *et al.* (2008)	Gamma
	Psychiatrist visits (ECT)	0.05	0.01	Knapp *et al.* (2008)	Gamma
	Psychiatrist visits (rTMS)	0.39	0.08	Knapp *et al.* (2008)	Gamma
	A&E visits (ECT)	0.02	0.003	Knapp *et al.* (2008)	Gamma
	A&E visits (rTMS)	0.02	0.004	Knapp *et al.* (2008)	Gamma
	GP visits (ECT)	0.97	0.19	Knapp *et al.* (2008)	Gamma
	GP visits (rTMS)	2.27	0.45	Knapp *et al.* (2008)	Gamma
Stable	Number of sessions (ECT)	0.25	0.05	Martínez-Amorós *et al.* (2012)	Gamma
	Number of sessions (rTMS)	0.25	0.05	Expert opinion	Gamma
	Psychiatrist visits	0.27	0.05	Pinto-Meza *et al.* (2008)	Gamma
	A&E visits	0.25	0.05	Expert opinion	Gamma
	GP visits	0.67	0.13	Pinto-Meza *et al.* (2008)	Gamma
	Clinical tests	0.21	0.04	Gothefors *et al.* (2010)	Gamma
Depression outpatient	Psychiatrist visits	0.54	0.11	Pinto-Meza *et al.* (2008)	Gamma
	A&E visits	0.50	0.10	Expert opinion	Gamma
	GP visits	1.34	0.27	Pinto-Meza *et al.* (2008)	Gamma
	Clinical tests	0.21	0.04	Gothefors *et al.* (2010)	Gamma
Depression inpatient	In-hospital (days)	7.48	1.50	Tafalla *et al.* (2010)	Gamma
	Psychiatrist visits	0.27	0.05	Pinto-Meza *et al.* (2008)	Gamma
	A&E visits	0.25	0.05	Expert opinion	Gamma
	GP visits	0.67	0.13	Pinto-Meza *et al.* (2008)	Gamma
	Clinical tests	0.21	0.04	Gothefors *et al.* (2010)	Gamma
	Hospitalization probability	0.13	0.03	Knapp *et al.* (2008)	Gamma
Unit costs	rTMS session	€319	€64	eSalud	Normal
	ECT session	€737	€147	HUC/eSalud	Normal
	In-hospital day	€369	€74	pestadístico	Normal
	Psychiatrist visit	€44	€9	eSalud	Normal
	A&E visit	€167	€33	eSalud	Normal
	GP visit	€21	€4	eSalud	Normal
	Clinical test	€12	€2	eSalud	Normal

	Using McSad (Bennett *et al.* 2000)		Using EQ-5D (Mann *et al.* 2009)		Probability distribution
	Value	s.e.	Value	s.e.	
Quality of life weights					
Acute Tx	0.090	0.020	0.519	0.290	Beta
Continuation Tx	0.590	0.018	0.645	0.230	Beta
Stable	0.790	0.023	0.759	0.250	Beta
Moderate depression	0.320	0.013	0.558	0.013	Beta
Severe depression	0.090	0.020	0.337	0.020	Beta

QALY, Quality adjusted life year; s.e., standard error; Tx, Treatment; ECT, electroconvulsive therapy; rTMS, repetitive transcranial magnetic stimulation; A&E, accident and emergency; GP, general practitioner; HUC, Hospital Universitario de Canarias; pestadítico, Ministry of Health Statistics website.

The number of sessions during a course of ECT and rTMS during acute treatment varies in clinical practice as well as in the trial protocols used in the studies identified. In the base case of our analysis we considered a mean of two ECT sessions per week and a mean of five rTMS sessions per week over the 2 weeks of acute treatment for each technique. This is reduced to one session every 15 days for ECT and two sessions every 15 days for rTMS during the continuation treatment. Once the patient has remitted he/she might continue with therapy to avoid re-occurrences; in those cases we consider one session every 2 months for both ECT and rTMS.

Over and above ECT and rTMS sessions, other healthcare resources used during the period of continuation of treatment were estimated based on the data collected in Knapp *et al.* (2008) on the number of inpatient stays, outpatient visits, GP visits and Accident & Emergency (A&E) attendances in each arm. The level of utilization of healthcare services in the depression and remitter states were extracted from studies detailing the use of healthcare consultations due to depression considering different states: stable, in-hospital with depression, out-of-hospital with depression (Pinto-Meza *et al.* 2008; Gothefors *et al*. 2010; Tafalla *et al.* 2010). The probability of hospitalization in this group of patients was estimated from Knapp *et al.* (2008). Based on communication with medical experts, we included an assumption on the number of A&E visits in the remission and depression periods.

Whenever available, unit costs were measured by the average cost of the Autonomous Communities in Spain that publish the official tariffs of the services of interest and were captured in the eSalud database (www.oblikue.com). We also used national data published on the Ministry of Health Statistics website (www.msn.pestadisitico.es). The unit costs of ECT was obtained from a cost analysis performed by the Hospital Universitario de Canarias detailing the healthcare resources (staff, material, equipment, tests, medication) used during an ECT session, to which we then applied national unit costs when those were available. Costs were expressed in 2013 euros (€); cost estimates reported for earlier years were inflated using the Consumer Price Index published at the National Institute of Statistics (INE; www.ine.es).

### QoL estimates

Bennett *et al.* (2000) estimated the value of the health-related QoL of patients with depression using a disease-specific utility measure (McSad) and by interviewing 105 patients with recent episodes of depression. McSad is a depression state classification system which consists of six dimensions: emotion, self-appraisal, cognition, physiology, behaviour, and role function, with four possible levels each. These data have been recently used in a cost-effectiveness analysis for the Health Technology Assessment programme on depression in UK (Greenhalgh *et al.* 2005). We used these data to estimate the QoL of individuals in each health state considered in our model (see Table 2). We also applied a different set of QoL values estimated in a study by Mann *et al.* (2009) on 114 patients with major depressive disorder participating in a RCT in the UK. Mann *et al.* used both the EQ-5D (www.euroqol.org) and SF-6D (www.shef.ac.uk/scharr/sections/heds/mvh/sf-6d), questionnaires which apply generic dimensions of health (i.e. mobility, self-care, usual activities, anxiety/depression, etc.) to characterize health-related QoL. They found that EQ-5D was more sensitive and able to capture changes in the participants, and therefore we considered the values estimated using EQ-5D in this study (Table 2). Not surprisingly, the disease-specific McSad instrument, which focuses on dimensions related to mental health, yielded lower QoL values in depression patients than the EQ-5D, which it is generally considered the preferred QoL instrument in economic evaluations (NICE, 2013), but that includes some dimensions not necessarily relevant for depressed patients. We report the cost-effectiveness results using both sets of utility scores (McSad and EQ-5D) separately.

### Probabilistic sensitivity analysis

In order to characterize the uncertainty in the model we undertook a probabilistic sensitivity analysis using Monte Carlo simulation. We applied probability distributions to each parameter which are summarized in Tables 1 and 2 and depend on the nature of the parameter (Briggs *et al.* 2006).

Probabilities were generally characterized by a beta distribution which is defined by two parameters, alpha and beta representing the occurrence and non-occurrence of an event, respectively. Using the data derived from the RCT that compared ECT and rTMS, we were able to construct relative risks for a series of events in our model. The logarithmic transformations of these relative risks were modelled using a normal distribution. Resource-use data inputs were characterized using a gamma distribution, while uniform distributions were applied to unit costs parameters; in both cases, we used upper and lower limits of 20% around the mean values with the exception of the number of sessions of both ECT and rTMS during continuation treatment for which we applied a 50% variation given the reported variation across studies about these input values. We used beta distributions to characterize the uncertainty around the utility values.

We applied 1000 simulations in the Monte Carlo analysis. For each simulation we obtained the mean cost and QALY of each alternative which we graphically represented in the cost-effectiveness plane. These simulations were also used to compute the CEAC, which represents the probability that each alternative is cost-effective at different values of willingness to pay per effectiveness unit.

## Results

The results of the analysis are presented in Table 3. The mean QALY value, using the utility weights as estimated by Bennett *et al.* (2000) are 0.263, 0.214, and 0.178 for the strategies rTMS followed by ECT, ECT alone, and rTMS alone, respectively. Using the utility scores reported in Mann *et al.* (2009) the QALYs for these treatment strategies are 0.460, 0.425, and 0.399, respectively. In both cases, rTMS + ECT achieved the higher QALY value, followed by the ECT-alone option, and last the rTMS-alone strategy. The mean cost over the 12-month interval of rTMS followed by ECT, ECT-alone, and rTMS-alone strategies are €20 279, €16 690, and €16 858, respectively. Therefore the use of ECT leads to the lowest estimated costs, while the strategy of using both rTMS and ECT when the former fails yields the largest mean costs.

**Table 3.** Expected cost and QALY results

	Cost (95% CI)	QALY (95% CI) using McSad	QALY (95% CI) using EQ-5D
ECT	€16 690	0.2137	0.4253
rTMS	€16 858	0.1783	0.3988
rTMS followed by ECT	€20 279	0.2631	0.4598
Incremental			
ECT *v.* rTMS	€−168 (€−4065 to 4294)	0.0354 (−0.0319 to 0.0912)	0.0265 (−0.0279 to 0.07423)
rTMS + ECT *v.* ECT	€3589 (€577 to 6664)	0.0494 (0.0183 to 0.1148)	0.0345 (−0.0094 to 0.1017)
ICER			
ECT *v.* rTMS		ECT alone dominates	ECT alone dominates
rTMS + ECT *v.* ECT		€72 668	€103 953

QALY, Quality adjusted life year; ECT, electroconvulsive therapy; rTMS, repetitive transcranial magnetic stimulation; ICER, incremental cost-effectiveness ratio; CI, confidence interval.

Combining the differences in costs and in QALYs we observed that the rTMS-alone strategy is dominated by the ECT-alone strategy, irrespective of the source of utility weights used, i.e. ECT alone leads to better medical results and is cheaper than using rTMS alone. On average, ECT yields to an effectiveness gain of 0.035 or 0.0264 QALYs, depending on the utility weights used, and is €168 less costly per patient in the year under analysis. When comparing use of rTMS followed by ECT *v.* ECT alone, we observed that the incremental cost of providing both treatment options when needed is €3589, while the incremental effectiveness is between 0.035 and 0.049 QALYs. The estimated incremental costs per QALY gained of the strategy rTMS + ECT compared with ECT alone are €72 668 and €103 953, which are in both cases considerably higher than the reference willingness-to-pay threshold recommended in this type of studies in Spain and other countries.

**Fig. 2.**
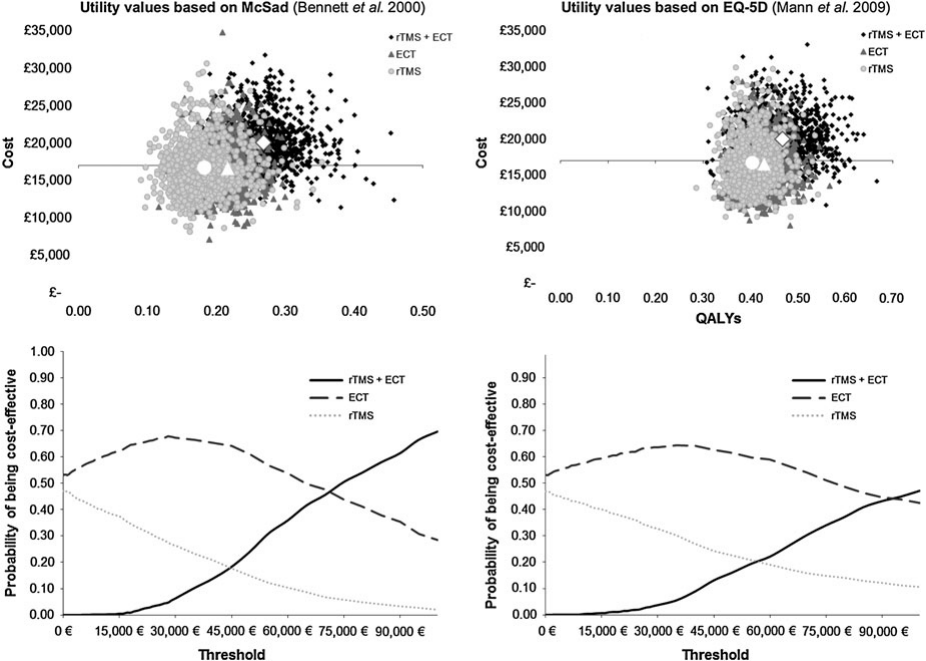
Monte Carlo simulation and cost-effectiveness acceptability curves. QALY, quality adjusted life year; ECT, electroconvulsive therapy; rTMS, repetitive transcranial magnetic stimulation.

Fig. 2 shows graphically the uncertainty with respect to the estimated costs and QALYs related to each strategy. The upper panels represent the pair of mean cost and QALY values of each of the Monte Carlo simulations for each of the three alternative strategies and under both sets of utility values. We observe that there is a large degree of overlapping between the strategies of providing ECT alone and rTMS alone, although for a proportion of simulations the ECT strategy is estimated to be more effective. The strategy of providing rTMS followed by ECT when rTMS fails is both more effective and more expensive in the majority of the simulation results. The lower panels show the CEACs. At the reference threshold value of €30 000 per QALY, the strategy with the highest probability of being cost-effective is the ECT-alone option with a probability of nearly 70% which is reduced to 63% when the utility scores used are those reported in Mann *et al.* (2009).

## Discussion

Depression places a huge burden in society both in terms of morbidity and mortality and also in terms of economic costs. In Spain the direct medical costs of depression and bipolar disorders have been estimated as €648 million in 2002 (López Bastida & Oliva Moreno, 2005). More recently and in Catalonia alone (with a population share of over 15% of the total Spanish population) the direct healthcare costs of depression were estimated as €156 million in 2009 prices (Salvador-Carulla *et al.* 2011). These high levels of public spending and the rising costs of treatments have intensified the need for information on the cost-effectiveness of interventions for depression (Barrett *et al.* 2005).

Although there are effective treatments for depression, such as antidepressant medication or psychotherapy, in severe cases ECT and rTMS can be considered as first- or second-line treatments. The British NICE guideline considers the use of ECT for the acute treatment of major depression that may involve a threat to life, for those cases requiring a rapid response, or when other treatments have failed (National Collaborating Centre for Mental Health, 2010). Regarding the safety of this technique, damage on several cognitive domains has been observed immediately after the treatment (Carney *et al.* 2003; Dunne & McLoughlin, 2012). Nevertheless, recovery of damaged domains, even the improvement in several domains in comparison to base values, is observed after a few days. With regards to rTMS, the NICE guideline concludes that there is no evidence suggesting major safety concerns, but there is uncertainty regarding the procedure's clinical efficacy. In sum, ECT has been found to be more effective on reducing depressive symptoms than repetitive rTMS, but the risk of adverse effects associated with rTMS is lower. This highlights the need to compare these treatment options in terms of the clinical effectiveness and potential side-effects, and, importantly, in terms of their cost-effectiveness.

Published evidence on the cost-effectiveness of ECT and rTMS is inconsistent. The conflicting conclusion in Knapp *et al.* (2008) might be due to the fact that their analysis was based on data from a single trial and did not include all available evidence. By contrast, the analysis by Kozel *et al.* (2004) can no longer be considered to be a complete picture of what is currently known about the cost-effectiveness of ECT compared to rTMS and hence requires updating with information from more recent trials. The aim of this study was to undertake an economic evaluation of the use of ECT alone, rTMS alone, and rTMS followed by ECT in the treatment of resistant severe depression based on all available evidence.

Our results are in line with the results found in Knapp *et al.* (2008). ECT alone was estimated to be the strategy more likely to be cost-effective; rTMS alone was found to be on average less effective and more expensive than ECT alone. In our evaluation we also compared ECT and rTMS alone with the option of providing rTMS followed by ECT when rTMS fails as in Kozel *et al.* (2004). Contrary to the results reported in Kozel *et al.* (2004), and after accounting for recent evidence, we found that this strategy was not likely to be cost-effective at standard values of willingness to pay per effectiveness unit.

Our study has a number of limitations. First, we have focused on the NHS perspective in Spain and thus we did not include patient and family costs nor did we account for productivity losses related to the condition. Labour productivity losses related to depression are significant and in Spain they were estimated at €1685 million in 2002 (López Bastida & Oliva, 2005). There is, however, controversy regarding the need to account for productivity losses on cost-effectiveness analysis, and the NICE guideline on technology appraisal explicitly indicates that productivity costs should not be included in either the reference or non-reference case of cost-effectiveness analyses (NICE, 2013). Second, given the paucity of evidence for longer periods, we considered a 12-month time horizon in the evaluation which might not fully capture the differences across strategies in the costs and effectiveness related to the interventions. However, previous studies have found that the scores in the HAMD are not different between intervention groups after 6 months of treatment (Knapp *et al.* 2008), which might suggest that the main differences are likely to occur within the time-frame of our analysis. Third, there might be other unmeasured factors that affect patients’ experience in the use of these techniques, such as fear or anxiety regarding the receipt of treatment, and especially for ECT. Furthermore, while there are a number of good quality studies on the effectiveness of ECT *v.* rTMS in the literature, the model developed for this analysis required information for a number of parameters which were not measured in these comparative studies such as suicide rates and the risk of discontinuation of treatment after remission which were estimated from the richer literature on the use of ECT. We also had to impose some assumptions in the model parameters, such as using some healthcare resource-use information from the detailed description recorded alongside a RCT undertaken in the UK, to which we applied unit costs related to the Spanish context. There might be regional differences on how care is delivered and thus we ran a deterministic sensitivity analysis to explore the impact of this assumption. We found that applying 50% upper and lower limits around each of the resource-use figures reported in the UK study did not impact on our results (see Table 4). The parameters that we found to have some impact on the results were the ECT and rTMS treatment costs, for which we found that in the case of acute rTMS treatment costs was 50% lower (or ECT costs 50% higher) while holding the remaining parameters constant, then the incremental cost per QALY gained of ECT alone compared to rTMS would be around €40000 (Table 4). Finally, it is worth mentioning that while in some cases we used data from international studies, a series of input values are specific to Spain, such as unit costs and some parameters on healthcare resource use. As a result, our conclusions might not be applicable to other healthcare contexts.

**Table 4.** Incremental cost-effectiveness ratio after one-way deterministic sensitivity analysis on selected parameters

	ICER: ECT *v.* rTMS		ICER: rTMS + ECT *v.* ECT	
Parameter	50% lower	50% higher	50% lower	50% higher
Continuation Tx				
In-hospital days ECT	ECT dominates	€1699	€72 881	€72 455
In-hospital days rTMS	€5737	ECT dominates	€65 149	€80 186
Psychiatrist visits ECT	ECT dominates	ECT dominates	€72 671	€72 665
Psychiatrist visits rTMS	ECT dominates	ECT dominates	€72 306	€73 030
A&E visits ECT	ECT dominates	ECT dominates	€72 672	€72 664
A&E visits rTMS	ECT dominates	ECT dominates	€72 603	€72 733
GP visits ECT	ECT dominates	ECT dominates	€72 695	€72 641
GP visits rTMS	ECT dominates	ECT dominates	€71 653	€73 683
Acute Tx				
ECT treatment costs	ECT dominates	€39 132	€73 599	€71 737
rTMS treatment costs	€41 969	ECT dominates	€39 202	€106 134

ICER, incremental cost-effectiveness ratio; ECT, Electroconvulsive therapy; rTMS, repetitive transcranial magnetic stimulation; Tx, treatment; A&E, accident and emergency; GP, general practitioner.

This evaluation shows that ECT is likely to be the most cost-effective option for the treatment of resistant severe depression given the estimated lower effectiveness of rTMS and the lack of evidence regarding cost savings related to the use of rTMS. The evidence provided in this study will be useful to inform decision makers considering information on relative effectiveness and costs when facing the choice between these alternative techniques.
